# Protective Relationships, Adultification, and Sexual Risk Among Black Adolescent Girls: Examining Maternal, Paternal, Peer, and Community Influences

**DOI:** 10.3390/ijerph23081039

**Published:** 2026-08-10

**Authors:** Natasha Crooks, Nyssa Snow Hill, Abigail Bushnell, Rabiatu Barrie, Stephanie Castelin, Gina Sissoko

**Affiliations:** 1 Department of Human Development Nursing Science, College of Nursing, University of Illinois Chicago, 845 S. Damen Avenue, Room 816, Chicago, IL 60612, USA; castelin@uic.edu; 2 College of Science and Health, Psychology, DePaul University, 1 E. Jackson Blvd., Chicago, IL 60604, USA; nsnow@depaul.edu (N.S.H.); abushnel@depaul.edu (A.B.); 3 Family Science Department, School of Public Health, University of Maryland, 4200 Valley Drive, Suite 2242, College Park, MD 20742-2611, USA; rbarrie@umd.edu; 4 Social, Behavioral Sciences and Center, Yale School of Public Health, 60 College Street, New Haven, CT 06520-0834, USA; ginadiagou.sissoko@yale.edu

**Keywords:** adultification, sexual risk, Black girls, protective factors

## Abstract

**Highlights:**

**Public health relevance—How does this work relate to a public health issue?**

- Black girls face sexual health disparities, placing them at significantly increased risk of HIV and sexually transmitted infections.
- This study suggests that peer friendships may be a powerful protective factor to mitigate sexual risk and adultification of Black girls.

**Public health significance—Why is this work of significance to public health?**

- Adultification creates bias, which hyper-sexualizes Black girls, increasing their vulnerability to early sexual debut, sexual violence, and lack of support, while hindering their ability to seek protection.
- Findings demonstrate how peer friendships partially mediated the relationship between adultification and sexual risk.

**Public health implications—What are the key implications or messages for practitioners, policy makers and/or researchers in public health?**

- Programs, policies, and interventions addressing Black girls’ sexual health should consider how peer relationships shape adolescents’ responses to adultification and promote peer environments that support healthy sexual development.
- Public health planning should engage peers to enhance protective factors to reduce their sexual risk.

**Abstract:**

The adultification of Black adolescent girls, viewing them as less innocent and more adult-like, has implications for their sexual and psychological well-being. Adultification creates bias, which hyper-sexualizes Black girls, increasing vulnerability to early sexual activity, sexual abuse, and lack of support, while hindering their ability to seek protection. However, protection from parents, peers, and communities may play a critical role in mitigating this risk for Black girls. The current study examined the role of maternal, paternal, peer friendships, and communal protective factors in mediating the association between adultification and sexual risk among Black girls. Girls’ responses (*N* = 575), 9–18 years old, were analyzed. Multiple linear regression analyses were conducted to estimate the mediating effects of protective factors (mother, father, friendship, and communal) on adultification and sexual risk. In separate mediation models, paternal relationships and friendship partially mediated the association between adultification and sexual risk. However, in a parallel mediation model that simultaneously examined maternal and paternal relationships, friendship, and community protection, friendship emerged as the only significant indirect pathway. Greater adultification was associated with stronger peer friendship, which in turn was associated with greater sexual risk. Programs, policies, and interventions addressing the sexual health of Black girls should consider how peer relationships shape responses to adultification and promote peer environments that support healthy sexual development.

## 1. Introduction

Black girls experience persistent sexual health disparities, including disproportionate rates of HIV and sexually transmitted infections (STIs) in the United States (U.S) [1,2,3]. These inequities often emerge during adolescence and can have long-term implications for physical health, psychological well-being, and sexual development.

Adultification is the premature exposure of children to adult-like roles and the social perception that they are more mature, knowledgeable, or responsible than their age warrants [4]. The adultification of Black girls is closely tied to objectification and reflects societal perceptions that Black girls are less innocent and more sexual than their peers [4,5,6]. Research suggests that Black girls experience adultification beginning in early childhood, which may increase their vulnerability to sexual exploitation, victimization, and sexual risk [4,7]. Justification of violence against Black women is also associated with greater endorsement of sexualized stereotypes among both Black men and women [8]. Despite growing recognition of adultification as a harmful sociocultural process, little research has directly examined its association with Black girls’ sexual risk.

### 1.1. Protecting Black Girls Against Sexualization

Protection refers to the ways in which Black girls or their social support systems prevent their early sexualization and its resulting sequelae (e.g., violence, exploitation, HIV/STI risk) [9]. Sexual protection for Black girls emanates from themselves or their social networks and community. Black girls often cite caregivers, peers, and community as their primary sources of protection [10,11,12,13]. Protection is described as comprising physical protection (e.g., against HIV/STIs and physical violence) and emotional protection (e.g., against damage to emotional well-being and self-esteem), and is provided through physical, verbal, and psychological strategies [9,14]. Physical protection involves setting boundaries around Black girls’ environment to protect them against vulnerable situations (e.g., prohibiting sleepovers, curfews), sexually explicit messaging (e.g., monitoring TV programming), and predators (e.g., teaching appropriate boundaries) [15]. Verbal protection involves sharing information about potential dangers and helpful strategies, while maintaining open communication about sex [14,16,17,18]. Psychological protection involves rejecting stereotyped messages by gaining information about sexuality, associating with peers who reject these messages, overcoming the culture of silence surrounding sex, and instilling positive values (e.g., self-worth, self-esteem, and confidence) within Black girls [14,19].

Although these strategies are often intended to promote safety, some may inadvertently increase risk. Sexual socialization characterized by silence, shame, restriction, or fear-based messaging may discourage Black girls from seeking information, discussing sexual concerns, communicating with peers or caregivers, or accessing sexual health services. Consequently, protective intentions do not always translate into protective outcomes. As such, understanding how individuals protect Black girls may help to identify appropriate targets for intervention.

### 1.2. Communal, Peer, and Maternal Relationships

Black girls’ communities, peers, and caregivers play important roles in shaping sexual development and sexual risk. Community environments characterized by violence exposure have been associated with increased sexual risk behaviors, whereas positive community influences, including mentorship and religious involvement, may serve protective functions [20,21]. Peer norms and relationships shape attitudes and behaviors related to dating, substance use, and sexual decision-making and have been associated with increased sexual risk among adolescents [22,23]. At the same time, peer relationships can provide important sources of social support, a sense of belonging, and identity development during adolescence [24].

Mothers or female caregivers remain among the most influential socializing agents in Black girls’ sexual development. Black girls receive messages about sexuality through their mothers, who often serve as primary protectors and sources of sexual socialization. Prior research suggests that maternal warmth, attachment, acceptance, and open communication about sexuality may promote healthier sexual development and lower sexual risk [12,22,25,26]. However, the effects of maternal communication appear to depend on the quality and content of those interactions [27]. Messages characterized by openness, affirmation, and developmentally appropriate guidance have been associated with positive outcomes, whereas silence, shame-based messaging, or restrictive communication may have unintended negative consequences [9,28]. Together, these findings suggest that communal, peer, and maternal relationships may shape how Black girls navigate sexual development and respond to experiences that place them at increased risk.

### 1.3. Paternal Protection

While the influence of community, peers, and mothers on Black girls’ sexual development has received greater attention in the literature, the role of Black fathers remains comparatively understudied. Emerging research suggests that Black fathers or male caregivers (e.g., brothers, cousins, grandfathers, and uncles) may serve as important sources of emotional support, guidance, and protection for Black girls [29,30,31,32]. Black girls have described male caregivers as providing physical protection, reinforcing self-esteem, promoting self-advocacy, and offering guidance about dating and relationships [30,31,32]. However, findings regarding the role of male caregivers in adolescent sexual development remain mixed, and relatively little research has examined these processes specifically among Black girls [33,34]. As a result, more research is needed to understand how paternal relationships may shape Black girls’ experiences of adultification and sexual health risk.

Examining paternal relationships alongside maternal relationships, friendship, and community protection may provide a more comprehensive understanding of the relational factors associated with adultification and sexual risk among Black girls. Although research has documented associations among adultification, sexual development, and relational protective factors, no studies have directly examined whether relational and community factors explain the link between Black girls’ experiences of adultification and sexual risk. Therefore, the present study examined maternal and paternal relationships, peer friendship, and community protection as potential mediators of the association between adultification and sexual risk among Black girls. We hypothesized that maternal and paternal relationships, peer friendship, and community protection would significantly mediate the link between adultification and sexual risk.

## 2. Methods

### 2.1. Becoming a Sexual Black Woman Framework and the Current Study

A life-course approach to examining health behaviors posits that factors across one’s life influence sexual development, ultimately shaping health and broader social inequities [35,36]. The Becoming a Sexual Black Woman framework provides a culturally grounded framework that describes factors impacting Black girls’ and women’s sexual and reproductive health [9]. In the framework, Black girls go through three developmental phases of sexual development—Girl, Grown, and Woman—that are influenced by sociocultural influences of stereotyped messaging and protection [9]. Understanding the people (i.e., parents, peers, and communities) who shape and protect Black girls’ sexual development and the messaging they receive may be helpful in finding ways to protect them from adultification and sexual risk. The Risk, Adultification, Messaging and Protection (RAMPs) scale [37], which was constructed from the Becoming a Sexual Black Woman Framework [9], measures how Black girls feel and how others interact with their bodies in relation to acting and looking grown, and how these experiences relate to their sexual risk. Guided by the Becoming a Sexual Black Woman framework [9], we explored whether peer, maternal, and paternal relational factors function as protective mechanisms linking adultification to sexual risk.

### 2.2. Participants

There were 956 participants originally recruited for this study. Participants were removed from the dataset if they opened the link but did not start the survey, resulting in 667 participants. Five participants were removed due to providing celebrity names (*n =* 662). Participants were excluded if they completed the survey in <7 min. The seven-minute threshold was established based on pilot testing and estimates of the minimum time required to thoughtfully read and respond to all survey items. One participant was removed because they did not answer any of the test items for the proposed measure (*n =* 579), and an additional four participants were removed because they were missing 75% of all the test items (*n* = 575). All participants identified as female and Black. Of the 575 participants, 83 were aged 9–12, 178 were aged 13–15, and 312 were aged 16–18.

### 2.3. Procedures

A pool of 38 items was generated from qualitative interviews with 25 Black girls who participated in a study on Black girl development, aiming to accurately capture phases of sexual development (i.e., looking, acting, and being grown; protection; and stereotype messaging) [38]. We then conducted cognitive interviews with a different set of 20 Black girls to further refine and consult on the measurement items [39,40]. The resulting items reflected the theoretical model developed from these analyses [9] and used wording that participants frequently used. An additional 9 items were added based on girls’ feedback, bringing the total to 43. These items were then evaluated in a REDCap survey with a larger sample of Black girls.

The present study represents a secondary analysis of data originally collected to develop and validate the Risk, Adultification, Messaging, and Protection Scale (RAMPS) [37]. Whereas the original study focused on scale development and psychometric evaluation, the current analyses examined adultification as a predictor of sexual risk and explored relational and community factors as potential mediators. Two recruitment methods were used: (1) community engagement, including snowball sampling through schools and community organizations, and (2) social media advertising. Initial recruitment efforts focused on community engagement in partnership with organizations that serve Black girls and families, including UI Health, Girls Inc. Chicago, and the Boys and Girls Club. Study flyers were distributed through community partner facilities, newsletters, websites, and stakeholder networks. Despite these efforts, recruitment through community partners yielded only 53 completed responses over an eight-month period.

To improve enrollment, recruitment was expanded to include paid Facebook and Instagram advertisements, as use of these platforms has increased among adolescents and their parents [41,42]. Social media advertisements were designed to appeal to both Black girls and their caregivers and were disseminated nationally. Advertisements invited interested individuals to learn more about the study through a secure survey link. Because parental consent was required for participants younger than 18 years of age, advertisements were intended to be viewed by both adolescents and their caregivers. Parents or caregivers who viewed the advertisements could share study information with eligible girls, and adolescents who encountered the advertisements were directed to involve a parent or caregiver in the consent process. Recruitment through social media enabled the study team to reach the target sample within approximately one month. Recruitment occurred nationwide over a 10-month period. Given the use of community-based, snowball, and social media recruitment methods, the final sample should be considered a convenience sample rather than a representative sample of Black girls in the U.S. These methods were selected to maximize access to a geographically diverse population of Black girls but may have favored participation among families with internet access, social media engagement, and willingness to participate in research.

Eligibility criteria included self-identifying as a Black girl aged 9–18 years, being fluent in English (speaking and reading), and living in the U.S. Participating teen girls and their caregivers provided voluntary, informed electronic assent and consent. Girls aged 9–17 provided an email address or phone number for their parents to receive REDCap links to review study information and provide electronic parental permission. After parental permission was received, girls then completed electronic assent. Girls aged 18 reviewed the study information, completed a brief screening questionnaire, provided electronic consent, and completed the survey. Girls and their parents were assured that the information provided would be kept confidential. Participants were asked to complete a survey in English that took approximately 15 to 20 min and received a $15 Amazon e-gift card for participating. All procedures were approved by the institutional review board at the University of Illinois Chicago.

### 2.4. Measures

**Risk, Adultification, Messaging, and Protection Scale (RAMPS).** The RAMPS is a 12-item measure assessing sociocultural factors that shape Black girls’ sexual development, including adultification, protection, and media messaging [37]. The scale includes three subscales: Adultification (4 items), Protection (4 items), and Messaging (4 items). Items are rated on a 5-point scale, with higher scores indicating greater endorsement of each construct; for the total score, adultification items are reverse-scored to reflect overall sexual risk protection. For the purposes of this study, we used only the Adultification subscale. Internal consistency for the adultification subscale was good (α = 0.81).

**Adolescent Clinical Sexual Behavior Inventory-Self Report** (ACSBI). The ACSBI-S is a 45-item self-report measure assessing adolescents’ sexual risk behaviors over the past 12 months [43]. Items are rated on a 3-point scale ranging from not true to very true. Friedrich et al. [43] reported strong internal consistency (α = 0.86). Internal consistency in the present sample was excellent (α = 0.95).

**NIH Toolbox Friendship Survey**. The NIH Toolbox Friendship Survey is a 5-item measure assessing youths’ perceptions of their friendships and social engagement [44]. Items are rated on a 5-point scale from never to always, with higher scores indicating a greater sense of connection and available peers with whom to interact. Internal consistency for the current sample was good (α = 0.80).

**NIH Toolbox Maternal Relationship Survey.** This 3-item scale assesses youths’ perceptions of closeness and meaningful time spent with their mother [45]. Two items use a 5-point response scale ranging from “never” to “always,” and one item uses a 4-point scale ranging from “extremely close” to “not very close.” Internal consistency in the present sample was acceptable (α = 0.77).

**NIH Toolbox Paternal Relationship Survey.** This 3-item scale parallels the maternal relationship measure and assesses youths’ perceptions of closeness and meaningful time spent with their father [46]. Two items are rated on a 5-point scale from *never* to *always*, and one item uses a 4-point closeness scale from *extremely close* to *not very close*. Internal consistency in the current sample was acceptable (α = 0.79).

**Community Protection**. Perceptions of community protection were assessed using a single item: “I feel protected within my community where I live (i.e., church, YMCA, youth group, organization).” Responses ranged from 1 (strongly disagree) to 5 (strongly agree), with higher values reflecting a greater sense of safety and protection within one’s community context.

### 2.5. Statistical Analyses

All analyses were conducted in SPSS Version 26, and mediation models were estimated using the PROCESS macro for SPSS Version 4.2 [47]. Data were examined both visually and statistically to assess normality. Missing data were minimal (0.03%) and addressed using mean imputation. While multiple imputations represent a more contemporary approach, given the negligible amount of missingness, mean importation was unlikely to meaningfully influence results. Composite variables were created by averaging corresponding items, with reverse coding applied where appropriate (e.g., maternal and paternal relationship items). Internal consistency for all multi-item scales was evaluated using Cronbach’s α, with values ≥0.70 considered acceptable.

Descriptive statistics were calculated for all study variables, and Pearson correlations were computed to examine bivariate associations. Age was included as a covariate in all subsequent analyses. Assumptions of linearity and homoscedasticity were met.

A multiple linear regression was conducted to test the effect of adultification on sexual risk. Separate exploratory mediation models were estimated for each potential mediator using PROCESS Model 4 [47]. Next, a parallel mediation model was estimated simultaneously examining maternal relationships, paternal relationships, friendship, and community protection as mediators of the association between adultification and sexual risk. Age was included as a covariate in all analyses. Mediation analyses were performed using bias-corrected bootstrapping with 5000 resamples to estimate indirect effects. Indirect effects were considered statistically significant when the 95% confidence interval did not include zero. Partial mediation was inferred when both the indirect effect (a*b path) and the direct effect (c′ path) were significant, whereas full mediation was indicated when only the indirect effect was significant. A two-tailed alpha level of 0.05 was used for all analyses.

## 3. Results

### 3.1. Preliminary Analyses and Main Effects

Pearson correlations were conducted to examine associations among study variables (see Table 1). As expected, adultification was positively associated with sexual risk (*r*(573) = 0.34, *p* < 0.001). Age was also significantly associated with sexual risk (*r*(573) = 0.24, *p* < 0.001) and was included as a covariate in all subsequent analyses. A multiple linear regression examined the effect of adultification on sexual risk. The overall model was significant, *F*(2, 572) = 58.35, *p* < 0.001, accounting for 17% of the variance in sexual risk (*R*^2^ = 0.17). Higher levels of adultification predicted greater sexual risk.

### 3.2. Mediating Roles of Maternal and Paternal Relationships, Friendship, and Community

**Figure 1:**
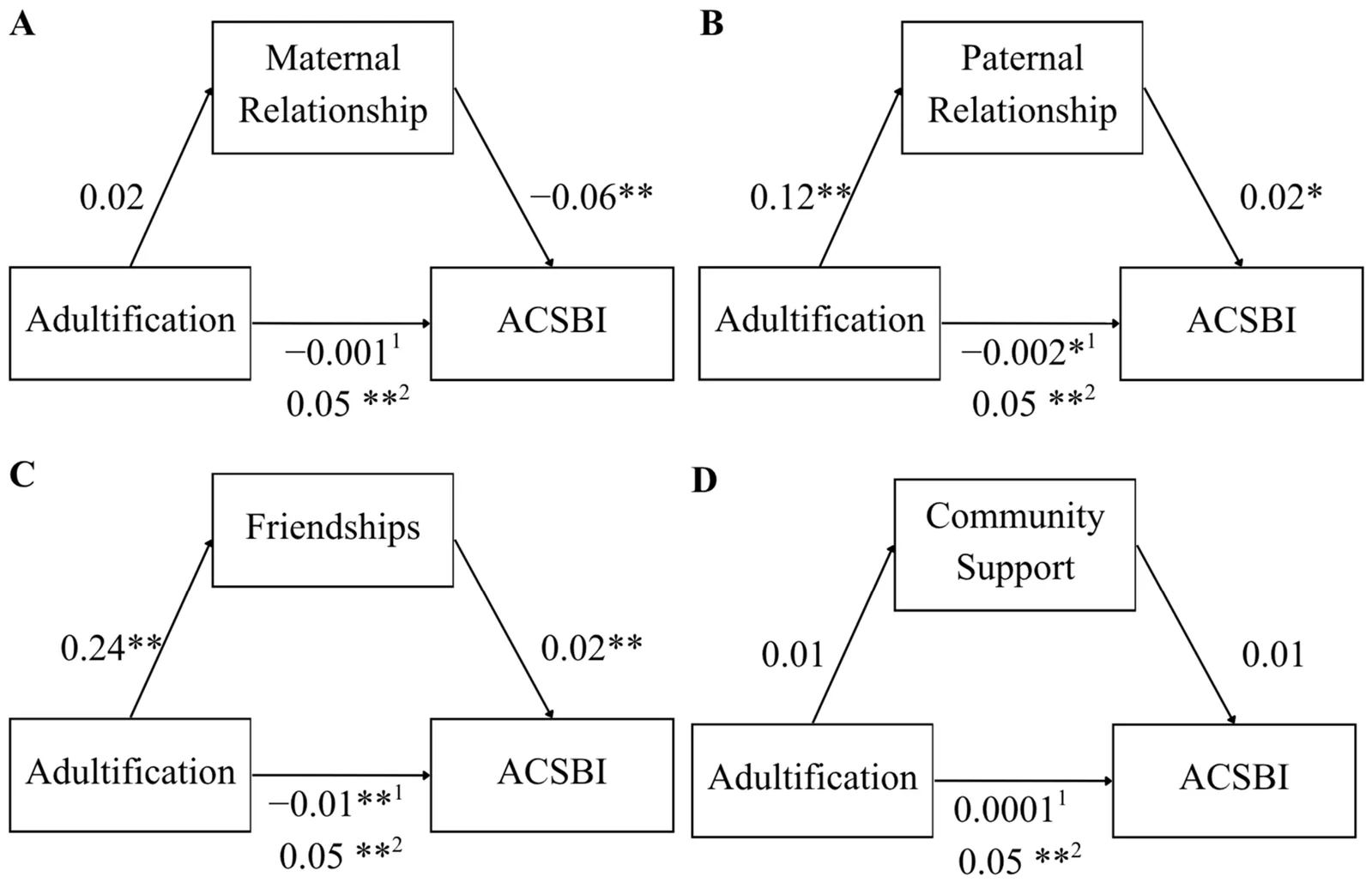
Separate models evaluating each relational mediator between adultification and sexual risk. Note. Four separate mediation models evaluating relational mediators between adultification and sexual risk while controlling for age. Panels represent unique mediators: (**A**) Maternal Relationship, (**B**) Paternal Relationships, (**C**) Friendships, and (**D**) Community Support. * *p* < 0.05, ** *p* < 0.01, ^1^ Indirect effect, ^2^ Direct Effect, ACSBI = Adolescent Clinical Sexual Behavior Inventory-Self Report.

First, separate mediation models were estimated for each relational mediator (see Figure 1). Maternal relationship did not significantly mediate the association between adultification and sexual risk (β = −0.00, 95% CI [−0.01, 0.00]). Similarly, community protection was not a significant mediator (β = 0.00, 95% CI [−0.00, 0.00]). Paternal relationships partially mediated the relationship between adultification and sexual risk. Mediation was supported by a significant indirect effect of adultification on sexual health risk (β = −0.002, 95% CI −0.004, −0.0002). Examination of the path coefficients showed higher levels of adultification were associated with better paternal relationships (β = 0.12, *p* = 0.002), and better paternal relationships were associated with decreased sexual risk (β = −0.02, *p* = 0.02). The direct effect of adultification on sexual risk remained significant, indicating partial mediation (β = 0.05, 95% CI 0.04, 0.06).

Friendship also partially mediated the relation between adultification and sexual risk. Mediation was supported by a significant indirect effect of adultification on sexual health risk (β = 0.01, 95% CI 0.002, 0.01). Higher levels of adultification were associated with higher levels of friendship (β = 0.24, *p* < 0.001), which, in turn, was associated with increased sexual risk (β = 0.02, *p* < 0.001). The direct effect of adultification on sexual risk remained significant, indicating partial mediation (β = 0.05, 95% CI 0.03, 0.06).

**Figure 2:**
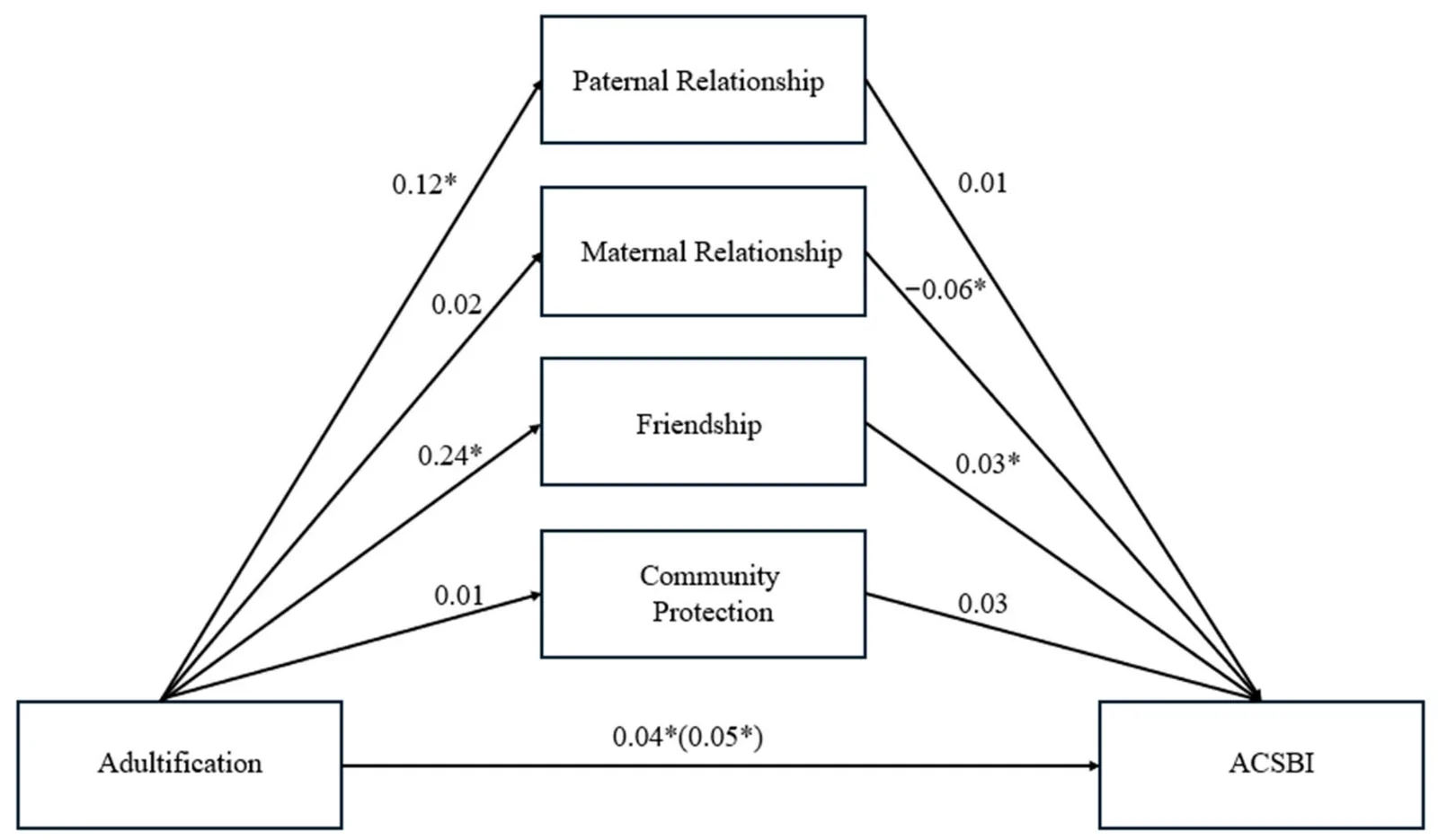
Parallel mediation model evaluating all relational mediators between adultification and sexual risk. Note. * *p* < 0.05, ACSBI = Adolescent Clinical Sexual Behavior Inventory-Self Report.

To account for overlap among protective factors, a parallel mediation model was estimated that simultaneously included maternal relationships, paternal relationships, friendship, and community protection as mediators while controlling for age (Figure 2). Adultification was significantly associated with paternal relationship quality (β = 0.12, *p* < 0.05) and friendship (β = 0.24, *p* < 0.05) but was not significantly associated with maternal relationship quality or community protection. When predicting sexual risk, friendship was positively associated with sexual risk (β = 0.03, *p* < 0.05), whereas maternal relationship quality was negatively associated with sexual risk (β = −0.06, *p* < 0.05). Neither paternal relationship quality nor community protection significantly predicted sexual risk. Age had a significant impact on sexual risk (*b* = 0.12, *p* < 0.01).

Examination of indirect effects indicated that friendship was the only significant mediator of the association between adultification and sexual risk (β = 0.01, *p* < 0.05). The direct effect of adultification on sexual risk remained significant, indicating partial mediation. Neither maternal relationships, paternal relationships, nor community protection demonstrated significant indirect effects.

## 4. Discussion

The present study examined whether maternal and paternal relationships, peer friendship, and community protection explained the association between adultification and sexual risk among Black girls. Consistent with expectations, higher levels of adultification were significantly associated with greater sexual risk, providing further evidence that premature positioning of Black girls as more mature, sexual, and responsible than their age leaves them vulnerable to harm [4,7]. However, when all four protective factors were examined simultaneously, peer friendship emerged as the only significant indirect pathway linking adultification and sexual risk. Peer friendship played a paradoxical role in the sexual risk of the Black girls who reported higher levels of being adultified. Closer peer friendships predicted greater sexual risk. These findings reify that adultification is not simply a perceptual bias but a factor with tangible outcomes and implications for the health and well-being of Black girls.

### 4.1. Adultification as a Sexual Risk Mechanism

The observed relationship between adultification and sexual risk aligns with the Becoming a Sexual Black Woman framework, which positions stereotyped messaging and premature characterization and imposition of adult roles as central to shaping sexual development [9]. Adultification appears to function as both an imposed and internalized process, wherein, because Black girls are treated as older and more sexually available, they internalize the messaging and adapt their behaviors in response to those characterizations and expectations. This dynamic likely accelerates their entry into sexually risky situations while simultaneously having limited access to developmentally appropriate guidance and protection [48]. This study provides evidence of the variable ways in which relationships with mothers, fathers, peers, and their communities interact within this strong predictive relationship between adultification and sexual risk and may offer pathways for intervention.

### 4.2. Maternal Relationship and Adultification

Maternal relationship quality was associated with lower sexual risk, suggesting an important protective role. This finding is supported by the literature demonstrating the importance of mother–daughter relationships as a protective factor [14,15,25,49,50]. However, because adultification was unrelated to maternal relationship quality, maternal relationships did not explain the association between adultification and sexual risk. This finding complicates narratives that position maternal relationships as uniformly protective [50,51,52]. We posit that general measures of closeness or perceived protection may not adequately capture the content or quality of gendered racial sexual socialization occurring within these relationships. As the prior literature suggests, Black maternal figures’ messaging and engagement with Black girls can be both protective and risk-inducing, particularly when they are characterized by silencing, shaming, or restriction rather than open, affirming, developmentally appropriate communication [38,53]. Thus, the absence of mediation is likely reflective of the variability in how protection is enacted in those relationships, rather than its absence altogether.

### 4.3. Paternal Relationship and Adultification

Contrary to our original hypothesis, paternal relationships did not emerge as a significant mediator in the parallel mediation model. However, adultification was positively associated with paternal relationship quality, such that girls reporting greater adultification also reported closer relationships with their fathers. When Black girls are perceived as older than they are, they may receive more emotional protection while simultaneously facing greater exposure to stereotypes that portray them as more sexually knowledgeable or responsible than their peers [4]. One possible explanation is that fathers may increase their engagement, guidance, reassurance, or affection when they perceive their daughters as vulnerable to early sexualization. Alternatively, girls experiencing adultification may seek greater support from fathers, or stronger father–daughter relationships may facilitate disclosure of adultification experiences. These findings align with the emerging literature highlighting the role of Black fathers in providing “insider” knowledge about male behavior, and the need to reinforce self-worth and offer relational protection [30,32]. It also suggests that paternal involvement may be particularly salient in contexts where girls are navigating early sexualization [54]. Black fathers provide their adolescent children with autonomy and agency in decision-making, taking on a more advisory role and trusting that their child will apply the lessons they have been given when faced with a warranted situation [55,56]. This parenting style can be edifying for Black girls, who often feel that they lack agency, particularly over their bodies [48,57]. Although paternal relationship quality was positively associated with adultification, paternal relationships did not uniquely explain sexual risk when maternal relationships, friendship, and community protection were considered simultaneously. These findings align with emerging scholarship emphasizing that the impact of adultification influences how caregivers perceive, respond to, and support Black girls during critical periods of development.

### 4.4. Peer Friendship and Adultification

The impact of peer friendship aligned with our hypothesis, which suggested it would significantly affect adultification and risk. Friendship partially mediated the relation between adultification and sexual risk. Higher levels of adultification were associated with stronger peer friendships, which in turn predicted increased sexual risk. These findings underscore the powerful role of friendships in shaping adolescent behavior and suggest that peer relationships may represent an important social context linking adultification and sexual risk. Girls experiencing adultification may seek support from peers with similar experiences; peer relationships may reinforce attitudes and behaviors associated with adultified expectations, or both friendship and sexual risk may reflect broader shared sociocultural influences. Because the present study was cross-sectional and friendship was measured as general connectedness rather than peer norms or sexual socialization, future research is needed to clarify the mechanisms underlying this relationship.

Developmentally, this is what we would expect. Adolescents, by and large, look to their friendships for validation and behavioral cues [24]. Adultification may influence the kinds of friendships Black girls have, and those friendships, in turn, help explain why adultification is associated with increased sexual behaviors. However, friendships do not tell the whole story. Adultification still has a direct effect, meaning other factors besides friendships also contribute to sexual risk. For Black girls experiencing adultification, girls within shared friendship networks may experience similar social pressures, including exposure to adultifying messages and expectations [24,58]. Thus, the protective efficacy of friendship networks depends on the norms and behaviors they reinforce. This finding highlights the importance of targeting peer friendships and networks in intervention efforts, with a focus on reeducation that shifts norms and develops skills to foster healthy, empowered sexual development.

### 4.5. Community Protection

Contrary to our hypothesis, community protection did not have any significant impact on adultification or sexual risk. The non-significant role of community protection suggests that feeling safe in one’s immediate environment does not necessarily translate into protection from sexual risk. Community-level safety may reduce exposure and vulnerability to violence broadly but may not directly interrupt the interpersonal and sociocultural processes through which adultification and sexual risk interact [59]. It also raises questions about whether a single-item measure can fully capture the multidimensional nature of community protection, particularly in contexts where opposing cultural messaging demanding protection of Black girls while simultaneously perpetuating those characterizations that increase their vulnerability to sexual harm coexist [60,61]. However, it is important to note that community protection was assessed with a single item, which likely does not capture its multidimensional nature. Consequently, the null findings may reflect measurement limitations rather than the absence of a meaningful relationship. Future research would benefit from the development of a more comprehensive measure of community protection.

### 4.6. Implications for Future Research and Practice

Black girls need and deserve protection. These findings suggest that protection is not a monolithic construct, but rather a dynamic process that varies across relational contexts. Interventions aimed at reducing sexual risk among Black girls must move beyond generalized notions of support and instead focus on how protection is enacted. This includes promoting open, culturally and developmentally appropriate sexual communication within families, particularly among mothers, and affirming and empowering fathers. Although paternal relationships did not uniquely mediate sexual risk in the final model, fathers and other male caregivers remain important relational supports in the lives of many Black girls. Future research should continue to examine how paternal involvement contributes to healthy sexual development and how fathers interact with broader family and peer systems that shape adolescent development. The findings also draw our attention to the need to address youth identity and how it informs peer friendships and norms that reinforce early sexualization and risky sexual behaviors. This may be addressed through culturally responsive school-based interventions, including topics such as adultification, sexual stereotypes, and peer influence that help Black girls recognize and challenge harmful societal narratives. The findings also call for greater attention to adultification as a significant risk mechanism. Prevention efforts that do not explicitly address the sociocultural forces that adultify Black girls may fall short, as they fail to intervene at the level where risk is introduced. Integrating critical consciousness, positive media engagement, literacy, and identity-affirming approaches may be particularly important in disrupting these processes.

### 4.7. Limitations

Study findings should be interpreted in the context of their limitations. The present study is a secondary analysis of data originally collected for scale validation. There are some sampling limitations. The sample was recruited primarily through social media advertisements, potentially limiting representation of more socially marginalized populations (i.e., Black girls without reliable internet access or whose caregivers do not use social media platforms) and therefore limiting the generalizability of the findings. Although recruitment advertisements were viewed by both parents and adolescents, parental consent was required for all participants under age 18. It may be that families with stronger parent-adolescent communication or greater parental engagement were more likely to participate, potentially introducing self-selection bias. The recruitment strategy was nationwide without a defined sampling strategy, which limits the ability to assess representativeness relative to a specific population. The original study was designed primarily for scale development and did not collect detailed information regarding socioeconomic status, family structure, geographic region, or urbanicity. Thus, the sample should not be considered representative of all Black adolescent girls in the U.S. We recommend that future research studies assess additional sociodemographic variables to better understand the sample’s representativeness.

Although mediation analyses were used to evaluate indirect associations consistent with theory, the data are cross-sectional and therefore cannot establish temporal or causal ordering. Adultification, relational factors, and sexual risk may influence one another reciprocally over time. Longitudinal research is needed to determine developmental sequencing and causal pathways. The focus on these three specific dimensions of parental protective factors (closeness and time spent) does not fully capture parents’ attitudes, values, or concerns regarding specific behavioral and psychosocial outcomes, nor does it account for the influence of sociocultural context. Relationship measures assessed only closeness and time spent together and did not capture the content or quality of protective communication. Additionally, the measures used in this study are limited by the number of items. For example, community protection was measured with a single item, which likely does not capture its multidimensional nature. Consequently, the null findings may reflect measurement limitations rather than the absence of a meaningful relationship. Measuring the nuances of adultification is particularly complex; however, no other psychometrically validated measure exists for this construct [37]. The Adultification subscale of the RAMPS is a newly developed measure that requires further validation by independent research teams.

## 5. Conclusions

This study examined the association between adultification and sexual risk among Black adolescent girls and explored relational and community mechanisms that may explain this relationship. Adultification was consistently associated with greater sexual risk. When maternal relationships, paternal relationships, friendship, and community protection were examined simultaneously, friendship was the only significant indirect pathway linking adultification and sexual risk. These findings highlight the importance of considering peer contexts when examining how adultification influences Black girls’ sexual development. Future research should further investigate the mechanisms through which peer relationships shape responses to adultification and identify intervention strategies that promote healthy, empowering, and developmentally appropriate sexual development for Black girls.

## Figures and Tables

**Table 1:** Descriptive Statistics and Correlations of Study Variables.

		*M*	*SD*	1	2	3	4	5	6	7
1.	Age	15.20	0.73	—						
2.	Friendship	18.79	3.68	0.15 *	—					
3.	Paternal	10.29	3.25	−0.14 *	0.12 *	—				
4.	Maternal	11.65	2.82	−0.15 *	0.12 *	0.44 *	—			
5.	Community	3.91	0.85	−0.07	0.22 *	0.13 *	0.23 *	—		
6.	Adultification	14.95	3.48	0.01	0.23 *	0.13 *	0.02	0.04	—	
7.	ACSBI	1.87	0.52	0.24 *	0.25 *	−0.08	−0.31 *	0.02	0.34 *	—

Note. Paternal = relationship with father, Maternal = relationship with mother, Community = community support, ACSBI = Adolescent Clinical Sexual Behavior Inventory-Self Report. * *p* < 0.05.

## Data Availability

The data that support the findings of this study are available from the corresponding author upon reasonable request.
